# The natural history of untreated small (< 5 mm) intracranial aneurysms: a five-year angiographic follow-up study

**DOI:** 10.1007/s10143-026-04507-3

**Published:** 2026-09-29

**Authors:** Jefferson Rosi, Juliana Gozzo Sekula, Leonardo B. O. Brenner, Manoel Jacobsen Teixeira, Saul Almeida da Silva, Jose Guilherme Mendes Pereira Caldas, Carlos Gilberto Carlotti Junior, Eberval Gadelha Figueiredo

**Affiliations:** 1 Department of Neurosurgery, Hospital das Clínicas, University of São Paulo, São Paulo, SP Brazil; 2 Department of Medicine, State University of Ponta Grossa, Av. General Carlos Cavalcanti - Uvaranas, Ponta Grossa - PR, Ponta Grossa, PR 84030-000 Brazil

**Keywords:** Small intracranial aneurysm, Natural history, Subarachnoid hemorrhage, Cerebral angiography, Cohort studies, Conservative management

## Abstract

The clinical management of small, unruptured intracranial aneurysms, particularly those measuring less than 5 mm, represents a significant area of debate in neurovascular surgery. The decision to pursue invasive treatment versus conservative surveillance is often guided by limited and sometimes conflicting evidence, leaving a critical gap in clinical decision-making. This study aimed to meticulously characterize the five-year natural history and morphological evolution of a cohort of patients with untreated small intracranial aneurysms (defined as < 5 mm), managed conservatively with serial angiographic surveillance. We conducted a longitudinal cohort study at a single tertiary academic center, enrolling 113 patients (101 female, 89.4%; 12 male, 10.6%; mean age 59 years) with at least one unruptured intracranial aneurysm measuring < 5 mm, diagnosed by digital subtraction angiography (DSA). All patients were managed with a conservative “watch-and-wait” protocol, which included a follow-up DSA at five years. The primary endpoints were morphological changes, including dimensional growth, shrinkage, complete resolution, de novo formation of new aneurysms, and the incidence of aneurysmal subarachnoid hemorrhage (SAH). Over the cumulative 565 patient-years of follow-up, no instances of aneurysmal rupture or SAH were observed. Most aneurysms were located in the anterior circulation (92.2%). The vast majority of aneurysms (n = 87; 77.0%) demonstrated complete morphological stability. A small subset of aneurysms exhibited dynamic changes: 12 (10.6%) demonstrated minor growth but remained within the < 5 mm size category, 6 (5.3%) decreased in size, and 5 (4.4%) underwent complete spontaneous resolution. De novo aneurysm formation was identified in 3 patients (2.7%). While traditional vascular risk factors like hypertension and oral contraceptive use showed no significant correlation with aneurysm behavior, a paradoxical and statistically significant association was found with smoking, which correlated with a higher likelihood of aneurysm resolution (p = 0.016). This study supports that a conservative management strategy with planned imaging surveillance is a safe and viable option for patients with unruptured intracranial aneurysms smaller than 5 mm. The absence of rupture over five years, coupled with the low rate of significant growth, suggests that these lesions have a very low observed risk of rupture and morphological progression over five years. These findings support conservative management for most patients with UIAs < 5 mm, although treatment decisions should remain individualized according to patient- and aneurysm-specific risk factors.

## Introduction

The incidental discovery of unruptured intracranial aneurysms (UIAs) has become an increasingly common clinical scenario, driven by the widespread use and advancing resolution of non-invasive neuroimaging modalities such as magnetic resonance angiography (MRA) and computed tomography angiography (CTA) [1]. While the specter of aneurysmal subarachnoid hemorrhage (SAH)—a condition with profound morbidity and mortality—looms over every diagnosis, the decision to treat a UIA is far from straightforward. It requires a meticulous balancing of the estimated lifetime risk of rupture against the immediate and long-term risks of morbidity and mortality associated with neurosurgical or endovascular intervention [2].

This clinical equipoise is most pronounced for small aneurysms. Large-scale prospective studies, most notably the International Study of Unruptured Intracranial Aneurysms (ISUIA), have been instrumental in shaping our understanding of UIA natural history, concluding that aneurysms smaller than 7 mm located in the anterior circulation, in patients without a prior history of SAH, carry a very low annual rupture risk [3]. Subsequent data from the UCAS Japan Investigators further stratified this risk, confirming a similarly low risk for small aneurysms [4]. However, these landmark trials often group aneurysms into broad size categories, potentially diluting the specific behavior of the smallest lesions [5, 6]. The natural history of aneurysms at the lowest end of the size spectrum—those measuring less than 5 mm—remains inadequately characterized, leaving their management in a “gray zone” often dictated by institutional bias or individual physician preference rather than robust evidence [7, 8].

This study was conceived to address this specific knowledge gap. By focusing exclusively on a cohort of patients with untreated UIAs measuring < 5 mm, we sought to provide high-resolution data on their medium-term morphological behavior. We hypothesized that aneurysms < 5 mm follow an indolent five-year natural history with low growth and rupture rates, supporting the rationale for conservative management.

## Materials and methods

### Study design and patient population

We performed a longitudinal analysis of a prospectively maintained database at the Hospital das Clínicas of the University of São Paulo Medical School (FMUSP), a high-volume tertiary neurovascular center. We identified all patients who were diagnosed with at least one unruptured intracranial aneurysm with a maximum diameter of less than 5 mm between January 2010 and December 2015. The diagnosis was confirmed in all cases by digital subtraction angiography (DSA), considered the gold standard for cerebrovascular imaging. Inclusion criteria were: (1) age > 18 years; (2) presence of at least one UIA < 5 mm; (3) no prior history of SAH from the index aneurysm; and (4) availability for a five-year clinical and angiographic follow-up. Patients with fusiform, dissecting, or mycotic aneurysms, as well as those for whom intervention was planned, were excluded. Our sample size was determined by convenience.

### Data collection and risk factor assessment

Baseline demographic data, including age, sex, and race, were collected. A detailed history of major vascular risk factors was obtained through structured interviews and review of medical records. This included: smoking status (defined as current or former regular use), diagnosis of systemic arterial hypertension (defined as a formal diagnosis requiring regular antihypertensive medication), and, for female patients, any history of oral contraceptive use.

### Imaging protocol and endpoint assessment

All patients underwent an initial four-vessel DSA at the time of diagnosis. Aneurysm size was defined by the maximum diameter of the sac in any projection. A standardized conservative management protocol was adopted, involving annual clinical follow-up and a scheduled repeat DSA at five years post-diagnosis. No other imaging modality was utilized. The decision for a five-year imaging interval was based on an institutional consensus that sought to balance diligent surveillance with the minimization of procedural risks and radiation exposure associated with annual angiography.

Two independent neurosurgeons, blinded to the initial report, reviewed the follow-up DSA images and compared them to the baseline studies. Discrepancies were resolved by a third senior partner. The primary endpoints were any morphological changes, categorized as: (1) Stability: no change in size or morphology; (2) Growth was defined as a measurable increase (≥ 1 mm) in any baseline aneurysm dimension, including neck width or height; (3) Shrinkage was defined as a measurable decrease (≥ 1 mm) in any baseline aneurysm dimension, including neck width or height; (4) Resolution: complete disappearance of the aneurysm sac with no residual neck; (5)De novo formation: appearance of a new aneurysm at a previously normal vascular location; and (6) Rupture: clinical or radiographic evidence of SAH.

### Statistical analysis

Descriptive statistics were used to summarize the cohort’s characteristics. Categorical variables were presented as frequencies and percentages, while continuous variables were presented as means with standard deviations (SD) or medians with interquartile ranges (IQR). All inferential analyses were performed at the patient level, with each patient contributing a single observation. For patients harboring multiple aneurysms, classification was based on the overall aneurysm evolution during follow-up; specifically, patients were classified as having worsening disease if any aneurysm demonstrated growth or if a de novo aneurysm developed. Associations between baseline risk factors and aneurysm behavior (regrouped as “worsening” [growth or de novo formation] vs. “non-worsening/improving”) were assessed using chi-square or Fisher’s exact tests for categorical variables and Mann–Whitney U tests for continuous variables. Bivariate logistic regression was used to calculate odds ratios (OR) with 95% confidence intervals (CI). For the primary endpoint of aneurysmal rupture, the observed annual rupture rate was calculated based on person-time at risk and reported with an exact 95% confidence interval using the Poisson distribution. A p-value of < 0.05 was considered statistically significant. All analyses were performed using IBM SPSS Statistics for Windows, Version 22.0 [9, 10].

## Results

Our final cohort consisted of 113 patients who completed the five-year follow-up protocol. The population was predominantly female (101 patients, 89.4%) with a mean age at diagnosis of 59 years (range: 19–72 years). Most aneurysms were located in the anterior circulation (92.2%). The majority of patients were Caucasian (84.0%). At least one major vascular risk factor was present in approximately half of the cohort (50%). Specifically, 31.9% had a history of smoking, 38.1% were being treated for hypertension, and 32.6% of women reported past or current use of oral contraceptives. The primary clinical endpoint—aneurysmal rupture—was not observed in any patient during 565 patient-years of follow-up, corresponding to an observed annual rupture rate of 0.0%. Using the exact Poisson method, the upper limit of the two-sided 95% confidence interval for the annual rupture rate was 0.65% per patient-year, corresponding to an estimated upper 95% confidence limit of 2.62% for the cumulative 5-year rupture risk.

The vast majority of aneurysms (*n* = 87, 76.9%) remained completely stable in size and morphology on the five-year follow-up DSA. Dynamic changes were noted in the remaining 26 patients (23.1%). Among these, aneurysm growth was detected in 12 patients (10.6%); however, in all these cases, the growth was minimal, and the aneurysms remained within the small aneurysm (< 5 mm) category. Favorable changes were also observed: 6 aneurysms (5.3%) shrank in size, and 5 (4.4%) resolved completely. Finally, 3 patients (2.7%) developed a de novo small aneurysm at a different location.

When analyzing the association with risk factors, no statistically significant correlation was found between aneurysm behavior and hypertension (*p* = 0.111) or oral contraceptive use (*p* = 0.059). An exploratory association between smoking and aneurysm resolution was observed (*p* = 0.016). The prevalence of smoking was highest among patients whose aneurysms resolved (4 of 5 patients, 80%) and was absent in those who formed de novo aneurysms (0 of 3 patients). However, this finding was based on a very small number of events and should be interpreted cautiously. This counterintuitive finding is detailed in Tables 1 and 2 shows the size of the aneurysms; Table 3 shows the location of the aneurysms.

**Table 1:** Cohort characteristics and association with aneurysm behavior at 5 years

Variable	Total (*N* = 113)	Unchanged (*N* = 87)	Growth (*N* = 12)	Shrinkage (*N* = 6)	Resolution (*N* = 5)	New aneurysm (*N* = 3)	*p*-value
Sex							0.763
Female	101 (89.4%)	79 (90.8%)	10 (83.3%)	5 (83.3%)	4 (80.0%)	3 (100%)	
Male	12 (10.6%)	8 (9.2%)	2 (16.7%)	1 (16.7%)	1 (20.0%)	0 (0%)	
Smoking							0.016
No	77 (68.1%)	63 (72.4%)	5 (41.7%)	5 (83.3%)	1 (20.0%)	3 (100%)	
Yes	36 (31.9%)	24 (27.6%)	7 (58.3%)	1 (16.7%)	4 (80.0%)	0 (0%)	
Hypertension (HTN)							0.111
No	70 (61.9%)	57 (65.5%)	5 (41.7%)	5 (83.3%)	1 (20.0%)	2 (66.7%)	
Yes	43 (38.1%)	30 (34.5%)	7 (58.3%)	1 (16.7%)	4 (80.0%)	1 (33.3%)	
Oral Contraceptives (OCP)							0.059
No	79 (69.9%)	61 (70.1%)	7 (58.3%)	6 (100%)	2 (40.0%)	3 (100%)	
Yes	34 (30.1%)	26 (29.9%)	5 (41.7%)	0 (0%)	3 (60.0%)	0 (0%)	

Values are n (%). Likelihood-ratio test. *OCP*: Oral Contraceptives

**Table 2:** Size of aneurysms

Size	Number of aneurysms	%
Up to 1.9 mm	65	34.60
2 a 2.9 mm	78	41.50
3 a 3.9 mm	41	21.80
4 a 4,9 mm	04	2.1
Total	188	100

**Table 3:** Location of aneurysms

Artery	Cases	%
right middle cerebral artery	24	12.8
left middle cerebral artery	21	11.2
left internal carotid artery in the ophthalmic segment	16	8.5
right internal carotid artery in the ophthalmic segment	08	4.2
anterior communicating artery	14	7.4
left internal carotid artery in the posterior communicating segment	12	6.4
right internal carotid artery in the posterior communicating segment	06	3.2
right anterior cerebral artery	11	5.9
left pericallosal artery	08	4.2
right pericallosal artery	03	1.6
right internal carotid artery in the paraclinoid segment	08	4.2
left internal carotid artery in the paraclinoid segment	05	2.6
basilar artery	07	3.7
left internal carotid artery in the choroidal segment	06	3.2
right internal carotid artery in the choroidal segment	04	2.1
right internal carotid artery in the cavernous segment	05	2.6
left internal carotid artery in the cavernous segment	05	2.6
right internal carotid artery in the pituitary segment	03	1.6
left internal carotid artery in the pituitary segment	03	1.6
right internal carotid artery in the bifurcation segment	03	1.6
left internal carotid artery in the bifurcation segment	02	1.0
left superior cerebellar artery	03	1.6
right superior cerebellar artery	01	0.5
right posteroinferior cerebellar artery	02	1.0
left posteroinferior cerebellar artery	01	0.5
right posterior cerebral artery	01	0.5
left internal carotid artery segment of the cavum	01	0.5
right lenticulostriate artery	01	0.5
left lenticulostriate artery	01	0.5
right internal frontal artery	01	0.5
Total	188	100

## Discussion

This study provides a focused, high-resolution view into the five-year natural history of untreated intracranial aneurysms smaller than 5 mm. Our principal finding is the benign course these lesions follow, justified by the absence of observed ruptures during follow-up of 113 patients. This result lends direct evidence to support a conservative management strategy, challenging the practice of prophylactic intervention for this specific aneurysm sub-population. We acknowledge that a 1-mm threshold approaches the limits of measurement precision for very small aneurysms and therefore some observed dimensional changes should be interpreted cautiously.

The observation that over three-quarters of these small aneurysms remained morphologically identical after five years suggests a state of biological inertia. Even among the minority that exhibited growth, the change was largely inconsequential; no aneurysm surpassed the 5 mm threshold into a higher-risk category. This suggests that growth in these small lesions is typically indolent [11, 12].

Furthermore, the phenomenon of spontaneous regression—including complete resolution in 4.4% of cases—is a critical finding. This strongly suggests that the natural history of small aneurysms is not a unidirectional path toward growth and rupture. Instead, it is a dynamic process that can lead to favorable outcomes, likely mediated by mechanisms of intra-aneurysmal thrombosis and endothelial remodeling [13, 14].

Perhaps the most intriguing finding of our study was the paradoxical association between smoking and aneurysm resolution. Smoking is a well-established risk factor for aneurysm formation, growth, and rupture, primarily through mechanisms involving chronic endothelial injury, inflammation, and extracellular matrix degradation [15, 16]. Therefore, this finding should be interpreted with considerable caution, as it was based on a very small number of events and may represent a chance observation rather than a true biological association.

Nevertheless, several hypotheses may explain this paradox. From a pathophysiological perspective, the dual effects of smoking on vascular biology have been proposed [17]. Although chronic exposure promotes aneurysm pathogenesis, nicotine-induced hemodynamic changes and a prothrombotic state could, in selected low-flow aneurysms, facilitate intrasaccular thrombosis followed by thrombus organization and endothelial remodeling, potentially resulting in aneurysm regression or apparent resolution [18].

An alternative explanation is methodological rather than biological. Despite being the gold standard for cerebrovascular imaging, digital subtraction angiography has finite spatial resolution, particularly for lesions approaching 1–2 mm [19, 20]. Consequently, apparent aneurysm resolution may reflect limitations in detecting extremely small lesions rather than true disappearance. Smoking-related vasomotor changes, altered hemodynamics, or parent vessel remodeling could reduce aneurysm conspicuity on angiography, resulting in a phenomenon of apparent or “pseudo-resolution,” especially among aneurysms at the smallest end of the size spectrum (< 3 mm) [21–24].

Given the limited number of events, these explanations remain speculative. The observed association should therefore be considered hypothesis-generating and interpreted as an exploratory finding requiring validation in larger prospective cohorts.

This methodological consideration underscores the importance of advanced imaging techniques, such as high-resolution 3D rotational angiography or even intravascular imaging, for the definitive assessment of very small aneurysms in future studies [25]. Other high-resolution imaging techniques, including 7-T MRI and optical coherence tomography, could be considered in future investigations. The apparent smoking-resolution association should therefore be interpreted with extreme caution, as it may reflect the limitations of our imaging modality rather than a genuine biological phenomenon.

While the literature extensively documents the surgical and endovascular management of small UIAs [26–28], our findings provide a necessary counterpoint to immediate intervention. This study supports a more nuanced, individualized approach, suggesting that conservative management may be appropriate, potentially mitigating procedure-related morbidity and reducing the associated burden of healthcare costs without compromising patient safety.

The implementation of a 5-year follow-up interval was predicated on the characteristically indolent natural history of these lesions and the current lack of high-level evidence favoring more frequent surveillance. While our findings indicate a high degree of stability, we recommend that readers interpret these results with appropriate caution regarding the potential for interval progression. The timing of aneurysm growth or regression between baseline and five years could not be determined. Serial interval imaging may better characterize the temporal dynamics of aneurysm morphology. Further research involving larger, diverse cohorts is necessary to confirm these longitudinal outcomes and to definitively exclude the risk of subclinical radiographic progression between imaging time points.

## Limitations of the study

This study is not without its limitations. First, its single-center design may limit the generalizability of our findings to other populations with different genetic or environmental backgrounds. Second, while the data were collected from a prospectively maintained registry, the analysis is retrospective in nature, which can introduce selection bias. Third, the sample size of 113 patients, while substantial for such a specific cohort, provides limited statistical power to draw definitive conclusions about risk factor associations, particularly for rare events like resolution. Considering most of the patients were female, results may not be generalized to males. The paradoxical finding regarding smoking, for instance, is based on only five events. Therefore, this observation should be considered hypothesis-generating rather than evidence of a causal association. Fourth, the use of a fixed five-year interval for follow-up imaging provides a robust medium-term snapshot but does not inform on the lifetime risk of rupture, which remains a key question for younger patients. The objective is, therefore, to keep monitoring the progression of those lesions across the years, updating the understanding of the pathology management. Finally, relying solely on DSA, while the gold standard, involves small but non-negligible procedural risks and radiation exposure.

## Conclusion

In this dedicated study of intracranial aneurysms < 5 mm, a conservative strategy of watchful waiting with imaging surveillance was demonstrated to be safe over five years, with no ruptures and a low rate of meaningful growth. Our findings advocate for a paradigm shift away from routine prophylactic intervention for these small, incidental lesions. The decision to treat should be reserved for rare and highly selected cases, as the inherent risks of neurosurgical or endovascular procedures are difficult to justify when the natural history risk is minimal. Decisions should remain individualized according to aneurysm characteristics and patient-specific clinical risk factors. We recommend extending the follow-up of this cohort to 10 years to solidify these findings and provide a more definitive understanding of the long-term safety of this conservative approach.

## Data Availability

No datasets were generated or analysed during the current study.
